# The Role of Genetic Factors and Kidney and Liver Function in Glycemic Control in Type 2 Diabetes Patients on Long-Term Metformin and Sulphonylurea Cotreatment

**DOI:** 10.1155/2014/934729

**Published:** 2014-06-09

**Authors:** Jasna Klen, Katja Goričar, Andrej Janež, Vita Dolžan

**Affiliations:** ^1^General Hospital Trbovlje, Rudarska cesta 9, 1420 Trbovlje, Slovenia; ^2^Pharmacogenetics Laboratory, Institute of Biochemistry, Faculty of Medicine, University of Ljubljana, Vrazov trg 2, 1000 Ljubljana, Slovenia; ^3^Department of Endocrinology, Diabetes and Metabolic Diseases, University Medical Center Ljubljana, Zaloška cesta 2, 1000 Ljubljana, Slovenia

## Abstract

This study investigated the influence of genetic polymorphisms of metformin transporters on long-term glycemic control and lipid status in type 2 diabetes patients in the everyday clinical setting. In total 135 patients treated with combination of metformin and sulphonylurea for at least 6 months were genotyped for *SLC22A1* rs628031 and *SLC47A1* rs2289669 polymorphisms. Relatively good blood glucose control with median HbA1c 6.9 (6.4–7.6) % was achieved on prescribed metformin dosage of 2550 (2000–2550) mg per day. Only 28 (20.7%) patients experienced mild hypoglycemia events, while no severe hypoglycemia events were observed. Most patients had normal or mildly impaired renal function. Parameters indicating renal function were not correlated with fasting glucose, HbA1c, or lipid parameters. Rs628031 and rs2289669 had minor allele frequencies of 0.385 and 0.355, respectively, and were not associated with HbA1c levels. Rs628031 was marginally associated with risk for hypoglycemia events (*P* = 0.046; OR = 0.51; 95% CI 0.26–0.99), while significant correlation was observed between rs2289669 and total cholesterol levels (*P* = 0.018). In conclusion, in patients on long-term metformin and sulphonylurea combination treatment, metformin transporters polymorphisms do not play a major role in glycemic control; however, they may influence lipid status.

## 1. Introduction

Metformin has been most commonly used as first line therapy for treatment of type 2 diabetes (T2D) for decades, due to both its good antihyperglycemic effect and safety profile [1].

Metformin directly and indirectly inhibits gluconeogenesis and lipid and cholesterol biosynthesis in the liver. It enters hepatocytes via organic cation transporter 1 (OCT1) and decreases ATP production via inhibition of mitochondrial respiration chain. Increased AMP levels trigger activation of AMP-activated protein kinase (AMPK) resulting in decreased glucose production and inhibition of lipid synthesis and increased fatty acid oxidation in the liver. It also leads to increased glucose uptake in muscle and hepatic cells [2, 3].

A considerable interindividual variability in glucose lowering response to metformin was reported with reduction of hemoglobin A_1c_ (HbA1c) values ranging from 0.8% to 3%. Furthermore, less than two-thirds of patients respond adequately to metformin and achieve a desired fasting glucose level [4]. Both nongenetic and genetic factors may be determinants of metformin effect. Among nongenetic factors, renal and liver functions are probably the most important factors influencing metformin effect [5], while pharmacogenetic studies have been focusing on gene variants related to metformin pharmacokinetics [6, 7].

Metformin is not metabolized, so drug transporters have the major role in its pharmacokinetics [8]. Organic cation transporters 1 and 2 (OCT1, OCT2), encoded by* SLC22A1* and* SLC22A2* genes, are the major transporters involved in the intestinal absorption, hepatic uptake, and tubular reabsorption of metformin [7, 9, 10]. Multidrug and toxin extrusion protein 1 (MATE 1) encoded by* SLC47A2* participates in metformin excretion through the bile and urine and is also involved in pharmacokinetics of metformin [11–13].

Several variants resulting in amino acid substitutions were shown to reduce metformin uptake via OCT1 in cell-based models [6, 14, 15] and influence metformin pharmacokinetics [9, 16]; however, most of these variants are very rare not only in Caucasian populations but also in other races [5, 6, 17, 18].* SLC22A1* rs628031 p.Met408Val is the most common polymorphism in Caucasian populations [16, 18, 19]. Although it was not shown to alter OCT1 mRNA expression levels or the activity of the transporter, it was associated with clinical effectiveness of metformin [15, 19, 20].

The effect of metformin transporters on treatment outcome still remains contradictory and even GWAS studies found no significant associations between polymorphisms in genes coding for metformin transporters and metformin response [21].

The aim of this study was to investigate the influence of common genetic polymorphisms in two metformin transporters on glycemic control and lipid status in T2D patients with defined renal and liver function in the everyday clinical setting.

## 2. Patients and Methods

### 2.1. Patients

Our prospective study included patients with T2D treated with combination of metformin and sulphonylurea at the General Hospital Trbovlje, Slovenia, for at least 6 months. Diagnosis of type 2 diabetes was based on the World Health Organization/American Diabetes Association definition of diabetes [22, 23]. Patients with diabetes type 1, gestational diabetes, other types of diabetes, active cancer, heart failure New York Heart Association (NYHA) 3-4, cotreatment with corticosteroids or estrogens, conditions that can cause hyperglycemia, addiction to alcohol or illegal drugs, and dementia or severe psychiatric disorders were not eligible for the study.

The study was approved by the Slovenian Ethics Committee for Research in Medicine and conducted in accordance with the Declaration of Helsinki. Written informed consent was obtained from all the subjects.

Information on the history of diabetes, presence of arterial hypertension, hyperlipidemia and chronic microvascular and macrovascular diabetic complications, smoking status, and concomitant diseases was obtained from the interview at the inclusion in the study and from the medical records. All subjects underwent a physical examination; blood pressure, body height, and body weight were measured and the body mass index (BMI) was calculated.

Glucose-monitoring devices were provided to all the patients participating in the study and they were carefully instructed on their use. All the patients were asked to measure fasting blood glucose levels once per week and additionally at any signs and symptoms suggesting low blood glucose for three months [24].

In all the patients glucose, HbA1c, lipid profile, liver enzymes, urea, creatinine, and estimated glomerular filtration rate (eGF) were determined in a fasting blood sample. At the same visit, microalbuminuria and albumin/creatinine ratio were determined in urine samples.

HbA1c was determined using immunoturbidimetric haemolytic automated analysis of 10 *μ*L capillary blood on Hitachi 912 (Hitachi, Japan). All other laboratory parameters were measured using standard laboratory procedure in the biochemistry laboratory of the General Hospital Trbovlje, Slovenia.

Hypoglycemia was defined as symptoms suggestive of low blood glucose confirmed by self-monitored blood glucose measurement below 3.9 mmol/L. Hypoglycemia events were classified into five groups according to American Diabetes Association criteria: (1) severe hypoglycemia, (2) documented symptomatic hypoglycemia, (3) asymptomatic hypoglycemia, (4) probable symptomatic hypoglycemia, and (5) relative hypoglycemia [25, 26]. Severe hypoglycemia was defined as any episode requiring assistance of another party. Groups 2 to 5 were considered as mild hypoglycemia events. The target HbA1c was defined as value lower than 7.0%. Albuminuria was classified as normal (<20 mg/L), moderately increased albuminuria (20–200 mg/L), and severely increased albuminuria (>200 mg/L). eGF was calculated according to the MDRD formula [27] and kidney function was assessed in line with the revised chronic kidney disease classification [28].

### 2.2. Genotyping

For each patient, 3−5 mL of peripheral blood was collected into test tubes with EDTA. Plasma was separated and stored at −80°C, and the cellular part of the blood sample was stored for a short term at −20°C until DNA isolation. Genomic DNA was isolated from peripheral blood leukocytes using Qiagen FlexiGene kit (Qiagen, Hilden, Germany). Genotyping of* SLC22A1* (*OCT1*) rs628031 G>A (p.Met408Val) and* SLC47A1 (MATE1)* rs2289669 G>A polymorphisms was carried out using a fluorescence-based competitive allele-specific (KASPar) assay according to the manufacturer's instructions (KBiosciences, Herts, UK).

### 2.3. Statistical Analysis

Median and interquartile range were used to describe central tendency and variability of continuous variables, while frequencies were used to describe the distribution of categorical variables. Fisher exact test or nonparametric Mann-Whitney test was used to compare clinical characteristics between different patient groups. The chi-square test was used to assess deviation from Hardy-Weinberg equilibrium (HWE). Logistic regression analysis was performed to assess the effects of the investigated factors on the occurrence of hypoglycemia episodes. Nonparametric tests were used to determine the influence of polymorphisms on continuous parameters, while nonparametric correlations were used for determining correlations between two continuous parameters. Univariate general linear model was used for determining the combined effect of two categorical variables on normally distributed logarithmic values of cholesterol level. The level of statistical significance was set to 0.05. Data were analysed using IBM SPSS Statistics 19.0 (IBM Corporation, Armonk, NY, USA).

## 3. Results

A total of 135 patients (males and females) were receiving combination treatment with metformin and SU for a median (25%–75% range) of 5 (4–8) years. The characteristics of the patient group are shown in Table 1. The median age of the patients was 64 (59–70) years. In total 55 (40.7%) patients were moderately obese (BMI > 30), while 32 (17.0%) patients were severely obese with BMI > 35.

The average duration of diabetes in the study group was 11 (6–16) years and was significantly correlated with age (*P* = 0.007). The prescribed metformin dosage was 2550 (2000–2550) mg per day. On the prescribed treatment regimen most of the patients achieved relatively good blood glucose control with the average HbA1c 6.9 (6.4–7.6) % (Table 1). The median (25–75% range) fasting blood glucose levels were 7.5 (6.7–8.8) mmol/L. Only 28 (20.7%) patients experienced a total of 67 mild hypoglycemia events, while no severe hypoglycemia events were observed. Among all hypoglycemia events, 38 (56.7%) were documented as symptomatic hypoglycemia, 27 (40.3%) were asymptomatic hypoglycemia, and 2 (3.0%) were probable symptomatic hypoglycemia.

Most patients had normal or mildly impaired renal function. Regarding the eGF stage, 47 (34.8%) patients belonged to stage 1, 74 (54.8%) to stage 2, 13 (9.6%) to stage 3a, and 1 (0.7%) to stage 3b. In total 88 (65.2%) patients had normal albuminuria, 38 (28.1%) had moderately increased albuminuria, and 9 (6.7%) had severely increased albuminuria. Liver function was also normal in most of the patients (Table 1). Significant differences were observed between patients on or without statin treatment regarding total cholesterol (*P* = 0.016), LDL cholesterol (*P* = 0.001), and TAG (*P* = 0.029), but not for HDL cholesterol (*P* = 0.342) (Table 1).

We observed that age was significantly correlated with rates of glomerular filtration (*P* < 0.001), creatinine levels (*P* = 0.009), and urea levels (*P* = 0.001), while a significant correlation was observed between diabetes duration and glomerular filtration (*P* = 0.047) and creatinine levels (*P* = 0.001). Parameters indicating renal function were not correlated with fasting glucose, HbA1c, or lipid parameters. No other clinical parameters were associated with HbA1c, total cholesterol, or LDL cholesterol. Increased BMI was associated with increased gamma-glutamyl transferase (GGT) (*P* = 0.042) and increased TAG (*P* = 0.003). Increased TAG levels were also associated with increased ALT (*P* = 0.031). Significant correlations were also observed between increased age and decreased HDL levels (*P* = 0.020).

All the patients were genotyped for* SLC22A1* rs628031and* SLC47A1* rs2289669 polymorphisms and the respective genotype frequencies are shown in Table 2. Both polymorphisms were in HWE with minor allele frequencies (MAF) of 0.385 and 0.355, respectively.

*SLC22A1* rs628031 polymorphism was marginally associated with risk for hypoglycemia events (*P* = 0.046; OR = 0.51; 95% CI 0.26–0.99), while* SLC47A1* rs2289669 did not influence the risk for hypoglycemia events (*P* = 0.310) (Table 2). The results remained the same even after adjustment for renal function (*P* = 0.046 and *P* = 0.311 for rs628031 and rs2289669, resp.). The investigated polymorphisms were not associated with HbA1c levels (*P* = 0.756 and *P* = 0.222 for* SLC22A1* and* SLC47A1*, resp.) (Table 3). Adjustment for renal function did not affect the association between polymorphisms and HbA1c. Both polymorphisms were also not correlated with BMI, HDL cholesterol, LDL cholesterol, or TAG, while significant correlation was observed only between* SLC47A1* and total cholesterol levels (*P* = 0.018) (Table 4).

**Figure 1:**
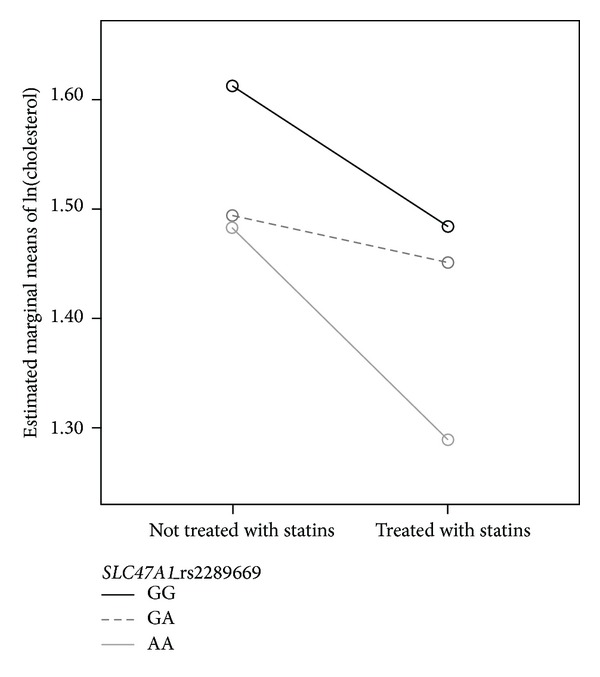
The combined effect of* SLC47A1* rs2289669 and statin treatment on logarithmically transformed values of cholesterol levels in T2D patients.

Because both* SLC47A1* rs2289669 genotype and statin use were associated with cholesterol levels, combined effect on cholesterol was examined using general linear model. As cholesterol levels were not normally distributed, logarithmically transformed values were used in the analysis. Both* SLC47A1* rs2289669 genotype (*F*(2,128) = 3.466; *P* = 0.034) and statin use (*F*(1,128) = 6.237; *P* = 0.014) remained significantly associated with cholesterol levels in T2D patients, treated with metformin, but the interaction was not significant (*F*(2,128) = 0.943; *P* = 0.392). Even if the results were adjusted for renal function,* SLC47A1* rs2289669 (*F*(2,127) = 3.383 and *P* = 0.037) and statin use (*F*(1,127) = 6.348; *P* = 0.013) significantly influenced cholesterol levels, while renal function was not significantly associated with cholesterol levels (*F*(1,127) = 0.540 and *P* = 0.464). Patients with polymorphic* SLC47A1* rs2289669 AA genotype who were treated with statins had the lowest cholesterol levels, while patients with* SLC47A1* rs2289669 GG genotype not treated with statins had the highest cholesterol levels (Figure 1).

## 4. Discussion

Our study was designed to investigate the influence of genetic polymorphisms of metformin transporters on glycemic control and lipid status in T2D patients with defined renal and liver function in the everyday clinical setting. Patients on long-term treatment were included in the study. This is a major difference in design from studies that investigated short-term response to metformin treatment, mostly including patients starting the treatment and evaluating response after a period of treatment [29–31]. T2D is a chronic metabolic disorder mostly requiring a life-long treatment, so it is of considerable interest to also study pharmacogenetic determinants of long-term treatment outcomes [24].

All patients were on metformin and sulphonylurea combination treatment. In general combinations of metformin and sulphonylurea are frequently used in the clinical practice as less than two-thirds of patients achieve the therapeutic goals on monotherapy with metformin [32]. Relatively good HbA1c levels were observed in our study group, although fasting blood glucose levels were mildly increased. As glucose control was not very tight, no severe and very few mild hypoglycemia events were observed despite sulphonylurea and metformin combination treatment. Although metformin does not cause hypoglycemia by itself in combination with sulphonylurea it may result in lower glucose levels and subsequently an increased risk for hypoglycemic events.

Our patient group was carefully checked for the kidney and liver function as metformin is primarily excreted via kidney and liver is its main target organ. Renal function was normal or mildly impaired in the majority of patients, so it was not expected that this could influence metformin pharmacokinetics. Population pharmacokinetic models have shown that metformin can be used also in patients with renal impairment with appropriate dosage adjustments based on the therapeutic drug monitoring [33]. As this is not routinely available in our country, patients with severe impairment of kidney function are not prescribed metformin; besides, this was an exclusion criterion in our study. Parameters indicating renal function were not correlated with fasting glucose, HbA1c, or lipid parameters. Metformin metabolic effects were also not influenced by liver function as it was mostly normal.

Although metformin reduces total and LDL cholesterol levels and increases HDL levels [34], placebo-controlled studies reported no significant effect of metformin treatment on total or HDL cholesterol; however, improvement in TAG levels was observed [3]. Similar to other studies [35], BMI and other clinical characteristics were not associated with glycemic control, total cholesterol, or LDL cholesterol. However, increased BMI was associated with increased TAG levels.

In our study group significant differences were observed between patients on or without statin treatment regarding total cholesterol, LDL cholesterol, and TAG, but not for HDL cholesterol. A placebo-controlled study in poorly controlled T1D patients also observed that metformin therapy significantly lowered total and LDL cholesterol independent of statin therapy [36].

Although some studies reported that* SLC47A1* rs2289669 genotype was significantly associated with the reduction in HbA1c after the first 6 months of treatment [30], we observed no influence of the studied metformin transporter polymorphism on long-term glycemic control. This is in agreement with other studies as most observed no association between metformin transporter polymorphisms and HbA1c levels [37].

Different polymorphisms in* SLC22A1* were either positively, negatively, or not correlated with treatment response in T2D [15, 16, 20, 31]; however, p.Met408Val polymorphism was found to be a positive predictor of metformin efficacy [20, 21]. Our data indicate that genetic variability of metformin transporters is not the major determinant of the long-term glycemic control, although they may contribute to metabolic effects of metformin treatment. In our study group the investigated* SLC22A1* polymorphism was not associated with HbA1c levels and only marginally associated with risk for hypoglycemia events, while significant correlation was observed between* SLC47A1* and total cholesterol levels.

In conclusion, we have shown that in patients on long-term metformin and sulphonylurea combination treatment polymorphisms of metformin transporters do not play a major role in glycemic control; however, they may influence lipid status. We have also observed an interesting correlation between* SLC47A1* and total cholesterol levels that was independent of statin treatment.

## Figures and Tables

**Table 1:** Patients' clinical characteristics.

	All patients (*N* = 135)	Not treated with statins (*N* = 47)	Treated with statins (*N* = 88)	*P*
Gender: male/female [number (%)]	78 (57.8)/57 (42.2)	27 (57.4)/20 (42.6)	51 (58.0)/37 (42.0)	1.000^a^
Age (years) [median (25%–75%)]	64 (59–70)	64 (57–70)	65 (60–70)	0.310^b^
BMI (kg/m^2^) [median (25%–75%)]	29 (28–34)	29 (28–32)	30 (28–34)	0.545^b^
BMI > 30 [number (%)]	55 (40.7)	16 (34.0)	39 (44.3)	0.274^a^
BMI > 35 [number (%)]	23 (17.0)	6 (12.8)	17 (19.3)	0.354^a^
Duration of diabetes (years) [median (25%–75%)]	11 (6–16)	11 (5–16)	10 (7–15)	0.967^b^
Duration of metformin treatment (years) [median (25%–75%)]	5 (4–8)	5 (3–8)	6 (4–8)	0.487^b^
Metformin dose (mg) [median (25%–75%)]	2550 (2000–2550)	2550 (1700–2550)	2550 (2000–2550)	0.202^b^
HbA1c (%) [median (25%–75%)]^d^	6.9 (6.4–7.6)	6.7 (6.2–7.7)	6.9 (6.5–7.6)	0.424^b^
Glucose (mmol/L) [median (25%–75%)]^e^	7.5 (6.7–8.8)	7.6 (6.7–8.8)	7.5 (6.7–8.8)	0.854^b^
Patients with hypoglycemia [number (%)]	28 (20.7)	7 (14.9)	21 (23.9)	0.269^a^
Serum creatinine (*μ*mol/L) [median (25%–75%)]	75 (64–87)	83 (64–91)	73 (63.3–83)	0.115^b^
eGF (mL/min) [median (25%–75%)]	82 (68–90)	78 (66–90)	84 (68.3–90)	0.473^b^
Urea (mmol/L) [median (25%–75%)]	5.4 (4.4–6.6)	5.3 (4.4–6.9)	5.4 (4.3–6.4)	0.872^b^
Albuminuria (mg/L) [median (25%–75%)]	9 (3.7–35.7)	7.1 (3.7–41.4)	9.5 (3.7–35.6)	0.642^b^
AST (*μ*kat/L) [median (25%–75%)]^c^	0.41 (0.35–0.50)	0.39 (0.35–0.51)	0.41 (0.35–0.50)	0.785^b^
ALT (*μ*kat/L) [median (25%–75%)]^c^	0.45 (0.36–0.62)	0.435 (0.36–0.54)	0.48 (0.35–0.68)	0.246^b^
GGT (*μ*kat/L) [median (25%–75%)]	0.42 (0.31–0.77)	0.42 (0.32–0.90)	0.42 (0.31–0.76)	0.621^b^
Total cholesterol (mmol/L) [median (25%–75%)]	4.3 (3.8–5.2)	4.8 (4–5.6)	4.1 (3.8–4.9)	0.016^b^
LDL cholesterol (mmol/L) [median (25%–75%)]	2.4 (1.9–3.3)	3 (2.1–3.6)	2.3 (1.9–2.9)	0.001^b^
HDL cholesterol (mmol/L) [median (25%–75%)]^c^	1.2 (1.0–1.4)	1.3 (1–1.5)	1.2 (1–1.4)	0.342^b^
TAG (mmol/L) [median (25%–75%)]	1.6 (1.3–2.4)	1.5 (1.1–2)	1.8 (1.3–2.7)	0.029^b^

^a^Calculated using Fisher exact test; ^b^calculated using Mann-Whitney *U* test; ^c^data missing for 1 patient; ^d^data missing for 3 patients; ^e^data for 88 patients (28 not treated with statins and 60 treated with statins).

**Table 2:** *SLC22A1* in *SLC47A1* genotype frequencies and the risk for hypoglycemia events.

Polymorphism	Genotype	All patients		Patients with hypoglycemia		
		*N* (%)	MAF	*N* (%)	*P*	OR (95% CI)
*SLC47A1* rs2289669	GG	54 (40.3)	0.355*	10 (18.5)	0.310	1.38 (0.74–2.59)
	GA	65 (48.5)		13 (20.0)		
	AA	15 (11.2)		5 (33.3)		

*SLC22A1* rs628031	GG	51 (37.8)	0.385	15 (29.4)	**0.046**	**0.51** ** (0.26**–**0.99)**
	GA	64 (47.4)		11 (17.2)		
	AA	20 (14.8)		2 (10.0)		

*Data missing for 1 patient; MAF: minor allele frequency.

**Table 3:** *SLC22A1* and *SLC47A1 *polymorphisms and HbA1c levels.

Polymorphism	Genotype	HbA1c median (25–75%)	*P* ^a^
*SLC47A1* rs2289669	GG	6.7 (6.4–7.5)	0.222
	GA	7.1 (6.5–7.7)	
	AA	6.7 (6.3–7.3)	

*SLC22A1* rs628031	GG	7.0 (6.4–7.4)	0.756
	GA	6.8 (6.4–7.7)	
	AA	6.8 (6.5–7.5)	

^a^Calculated using Kruskal-Wallis test.

**Table 4:** The influence of *SLC22A1* and *SLC47A1 *polymorphisms on BMI and lipid profile.

Polymorphism		BMI median (25–75%)	*P* ^a^	Total cholesterol median (25–75%)	*P* ^a^	HDL cholesterol median (25–75%)	*P* ^a^	LDL cholesterol median (25–75%)	*P* ^a^	TAG median (25–75%)	*P* ^a^
*SLC47A1* rs2289669	GG	30.0 (28.0–33.0)	0.835	4.6 (3.9–5.5)	**0.018**	1.2 (1.0–1.4)	0.683	2.7 (2.0–3.5)	0.070	1.7 (1.3–2.7)	0.562
	GA	29.0 (28.0–34.5)		4.5 (3.6–5.0)		1.1 (1.0–1.5)		2.4 (1.9–3.2)		1.6 (1.2–2.4)	
	AA	29.0 (26.0–35.0)		4.0 (3.4–4.2)		1.1 (1.0–1.3)		2.1 (1.9–2.5)		1.5 (1.4–1.9)	

*SLC22A1* rs628031	GG	29.0 (28.0–34.0)	0.387	4.5 (3.8–5.6)	0.711	1.2 (1.0–1.4)	0.096	2.5 (2.0–3.5)	0.365	1.5 (1.2–2.2)	0.569
	GA	30.0 (27.1–32.0)		4.4 (3.8–5.3)		1.2 (1.0–1.5)		2.4 (1.9–3.2)		1.8 (1.3–2.5)	
	AA	32.5 (28.3–34.0)		4.2 (3.7–4.9)		1.1 (0.9–1.3)		2.4 (1.7–3.1)		1.7 (1.2–2.9)	

^a^Calculated using Kruskal-Wallis test.
